# The use of oral contraceptive before pregnancy and breastfeeding duration: A cross-sectional study with retrospective ascertainment

**DOI:** 10.1186/1746-4358-3-29

**Published:** 2008-12-16

**Authors:** Nelís Soto-Ramírez, Wilfried Karmaus

**Affiliations:** 1 Epidemiology and Biostatistics Department, Arnold School of Public Health, University of South Carolina, 800 Sumter St, Columbia, SC, USA

## Abstract

**Background:**

Various studies have identified risk factors associated with decreased breastfeeding duration. The aim of this study was to investigate whether there is an association between oral contraceptive (OC) use before pregnancy and breastfeeding duration.

**Methods:**

In 1994/95, as part of a 3-year epidemiologic follow-up study of school children, reproductive interviews were conducted with their mothers. The study population consists of 663 women residing in Hesse, Central Germany; 575 provided information on their reproductive history. The interview included retrospective ascertainment of OC use, its timing before pregnancy, and duration of breastfeeding. To estimate its effect on duration of breastfeeding, survival analysis was applied controlling for maternal age, socio-demographic characteristics, smoking during pregnancy, age at menarche, planning of the pregnancy and birth order. Hazard ratios and median breastfeeding duration were estimated.

**Results:**

The mean age of the women at delivery was 27.3 years. Among participants, 34.9% had high school education or less, 10.4% had more than 2 children, and 30.1% smoked during pregnancy. In total, oral contraceptive use in the 12 months before conception was reported by 40.4% of the women, within 3 months of conception by 18.4%. 81.4% (468/575) of women initiated breastfeeding. Compared to those who did not use OC in the 12 months preceding pregnancy, mothers who used OC during the 3 months before conception had a shorter duration of breastfeeding (HR = 1.29; 95% CI: 1.03, 1.61), as did mothers who stopped OC use 4–12 months before conception (HR = 1.27, 95% CI: 1.02, 1.58). Smoking during pregnancy and lower education were also significantly associated with shorter duration of breastfeeding.

**Conclusion:**

The results suggest that OC use during the 12 months prior to conception may affect breastfeeding duration. These findings may be due to the endocrine disrupting effect of OC. Alternatively, both OC use and shorter duration of breastfeeding may represent lifestyle-related conditions.

## Background

Breastfeeding is the perfect way to nourish infants and protect them from illness [1]. Documented benefits to the nursing mother include a reduction in the risk of breast and ovarian cancer [2]. Other studies have identified various risk factors associated with a decreased duration of breastfeeding. Among those were smoking habits during pregnancy [3], Caesarean delivery [4,5], low socio-economic status [6,7], low maternal education [8], and employment [9].

In addition it has been reported that maternal burden of dichlorodiphenyldichloroethylene (DDE), a metabolite of the pesticide dichlorodiphenyltrichloroethane (DDT), is related to a shorter breastfeeding duration possibly by an endocrine disruptive effect [10-12]. An endocrine disrupter has been defined by U.S. Environmental Protection Agency as "an exogenous chemical substance or mixture that alters the structure or function(s) of the endocrine system and causes adverse effects at the level of the organism, its progeny, populations, or subpopulations of organisms, based on scientific principles, data, weight-of-evidence, and the precautionary principle" [[13], p. ES-1]. The finding that DDE was demonstrated to affect duration of breastfeeding raise the suspicion that oral contraceptives (OC), frequently used endocrine disruptors, may also be associated with breastfeeding duration. Indeed, studies have shown that OC use after pregnancy was related to a decreased duration of breastfeeding [4,14]. However, no study has yet examined the effect of OC use before pregnancy on length of breastfeeding. The objective of this study was to investigate whether use of OC before pregnancy reduces the total duration of breastfeeding.

## Methods

In 1994/95, a 3-year follow-up study of school children was initiated to monitor environmental health risk [15-19]. Parents of 1,091 second grade school children in 18 townships were invited to participate in this study. We obtained permits from the Data Protection Agency of the State of Hamburg, Germany; from the Ministry of Cultural Affairs of Hesse, Germany; and from the local school committees. Informed consent, according to the requirements of the Ethical Committee of the Board of Physicians and the Data Protection Agency of the State of Hamburg, was obtained from all participating parents. The study population consisted of 663 mothers of the index school children residing in Hesse, Central Germany. Women who adopted the index child were not interviewed about breastfeeding.

Trained personnel conducted face-to-face interviews with the mothers, retrospectively ascertaining health and living conditions. The interview questionnaire was based on the standardized questionnaire used in the European Studies on Fertility and Subfecundity [20]. The following variables were considered in this analysis: mother's age at delivery (< 20, 20–24, 25–29, ≥ 30 years); mother's education (high school or less, some college, and college graduate or higher); date of conception, date of delivery, outcome of index pregnancy; maternal age at menarche (≤ 11 years old, 12–15 years old, ≥ 16 years old); smoking habits during pregnancy (none, yes); planning this pregnancy (no, undecided, yes); and use of OC before pregnancy (none, yes), and its timing before pregnancy (0–3, 4–12 months before conception). Information on the type of OC (combined estrogen/progesterone or progesterone only) or its brand name was not collected. To determine birth order (one, two, three and more), the date of birth of each index child was positioned within the reproductive history of the mother. No mother reported a stillbirth before the birth of the index child. Regarding breastfeeding, we asked whether the child was breastfed, if so, the duration of exclusive breastfeeding (no other source of food) and the total duration of breastfeeding. The duration was collected in weeks. For descriptive purposes, breastfeeding duration was grouped into none, 0–2 months, 3–5 months, 6–12 months, and more than 12 months.

Proportions and average values were used for description. To estimate effects on duration of breastfeeding, we used proportional hazard regressions (survival analyses) to assess hazard ratios (HR) and their 95% confidence intervals. The HR reflects the relative risk of stopping breastfeeding conferred by each independent variable. The survival analyses also provided estimates of median breastfeeding duration for individual risk factors.

The risk factor of interest was OC use. To estimate HR, the following confounders ware taken into account: maternal age, socio-demographic characteristics, smoking during pregnancy, maternal age at menarche, planning this pregnancy and birth order. Since risk factors related to shorter duration of breastfeeding may change in the course of the reproductive history, we additionally stratified for birth order (one versus two and more).

Information on breastfeeding was collected only from women who breastfed. However, some mothers may have categorized themselves as non-breastfeeders if they had early difficulties and stopped breastfeeding in the few days following birth. We assumed that a combined analysis that included non-breastfeeding as zero duration in a time-to-stop breastfeeding analysis (survival analysis) may partially overcome this misclassification. However, in addition, we also tested the association of the risk factors for breastfeeding excluding non-breastfeeders. The SAS statistical package version 9.1 was used to conduct the data analysis [21].

## Results

In the index school children, 671 of 1,091 participated in the study (61.5%). A total of 663 women were interviewed; 639 provided information about their reproductive history including oral contraceptive use; 37 were not the natural mother of the index child and were not asked about breastfeeding. Of the remaining 602, information on breastfeeding duration was gathered from 575 mothers. In the following, we focused on total duration of breastfeeding. At delivery, the age range of women was 15 to 38 years with a mean age of 27.3 years (Table 1). Among participants, 34.9% had high school education or less, 10.4% had more than two children, and 30.1% smoked during pregnancy. Menarche before the age of 11 years was experienced by 13.5%. About half of the women who used OC were between 25 and 29 years old while 43.7% of the participants who used OC had some college education. In 62.8% of the OC users and 48.7% of the non-users, the index child was the first offspring. Smoking during pregnancy was reported in 37.6% of the OC users and 24.9% of the non-users. Among OC users 79.9% planned to become pregnant, 6.8% were undecided, and 13.3% did not plan to conceive (Table 1). Compared to users (79.9%), a lower proportion of non-users (68.8%) had a planned pregnancy.

**Table 1:** Study participants characteristics with and without oral contraceptive use in the 12 months before conception

	**No oral contraceptive use** **(n = 341)**		**Oral contraceptive use** **(n = 234)**	
	**N**	**%**	**N**	**%**
**Age at childbirth**				
< 20	10	2.9	5	2.2
20–24	67	19.7	82	35.0
25–29	146	42.8	104	44.4
≥ 30	118	34.6	43	18.4
				
**Education**				
≤ High school	113	33.6	86	37.1
Some college	134	39.9	114	49.1
≥ College graduate	89	26.5	32	13.8
Missing	5		2	
				
**Birth order**				
1	166	48.7	147	62.8
2	126	36.9	76	32.5
≥ 3	49	14.4	11	4.7
				
**Menarche**				
≤ 11 years old	38	11.3	39	16.7
12–15 years old	280	83.3	183	78.2
≥ 16 years old	18	5.4	12	5.1
Missing	5		0	
				
**Smoking during pregnancy**				
Yes	85	24.9	88	37.6
No	256	75.1	146	62.4
				
**Planned pregnancy**				
Yes	234	68.6	187	79.9
Undecided	35	10.3	16	6.8
No	72	21.1	31	13.3

Oral contraceptive use in the 4 to 12 months prior to conception was reported by 21.9% of the women, within 3 months by 18.4% (Table 2). Approximately 81% (468/575) of women initiated breastfeeding. In breastfeeding women who did not use OC, the median duration of breastfeeding was 14 weeks. In comparison, the median breastfeeding duration was 12 weeks in women who used OC in the 4 to 12 months before conception, and 8 weeks in those who used OC in the three months prior to conception. Longer breastfeeding was reported for women who were older at the birth of their index child. Also higher maternal education was related to longer breastfeeding. Smoking during pregnancy was related to a higher proportion of non-initiating breastfeeding (25.4% vs. 15.7%). Among breastfeeding women, those who smoked during pregnancy had a shorter duration of breastfeeding (median 8 weeks) than those that did not smoke during pregnancy (15 weeks). Approximately 24% of women with high school or less reported no breastfeeding initiation versus 7.4% with at least college education. Breastfeeding duration was comparable for those who planned to become pregnant (12.0 weeks) and those who did not plan the pregnancy (12.0 weeks). About 18% of women who planned their pregnancy reported no breastfeeding initiation and 20.4% who did not plan to become pregnant (Table 2).

**Table 2:** Proportions of no breastfeeding and median duration of breastfeeding

	**Total**	**Women not breastfeeding**		**Women breastfeeding**	
	**N**	**n**	**%**	**n**	**Median duration in weeks (5%, 95% value)**
**Oral contraceptive use before pregnancy**					
No	343	59	17.2	284	14 (2, 72)
0–3 months	106	18	17.0	88	8 (2, 48)
4–12 months	126	30	23.8	96	12 (2, 50)
					
**Age of the mother at birth**					
< 20 years	15	3	20.0	12	8 (3, 28)
20–24 years	149	26	17.4	123	9 (2, 42)
25–29 years	250	46	18.4	204	12 (3, 56)
≥ 30 years	161	32	19.9	129	16 (2, 78)
					
Mother education *					
≤ High school	199	48	24.1	151	8 (4, 78)
Some college	248	48	19.3	200	12 (2, 52.5)
≥ College graduate	121	9	7.4	112	24 (4, 78)
					
**Birth order**					
1	313	50	16.0	263	12 (2, 52)
2	202	42	20.8	160	15 (2, 75)
≥ 3	60	15	25.0	45	15 (2, 78)
					
Menarche *					
≤ 11 years old	77	22	28.6	55	12 (1, 52)
12–15 years old	463	80	17.2	383	12 (2, 56)
≥ 16	30	5	16.7	25	15 (2, 78)
					
**Smoking during pregnancy**					
Yes	173	44	25.4	129	8 (2, 50)
No	402	63	15.7	329	15 (2, 60)
					
**Planned the pregnancy**					
Yes	421	76	18.1	345	12.0 (2, 56)
Undecided	51	10	19.6	41	12.0 (2, 45)
No	103	21	20.4	82	12.0 (2, 56)

* Missing values are not shown for maternal education (7) and menarche (5)

**Figure 1:**
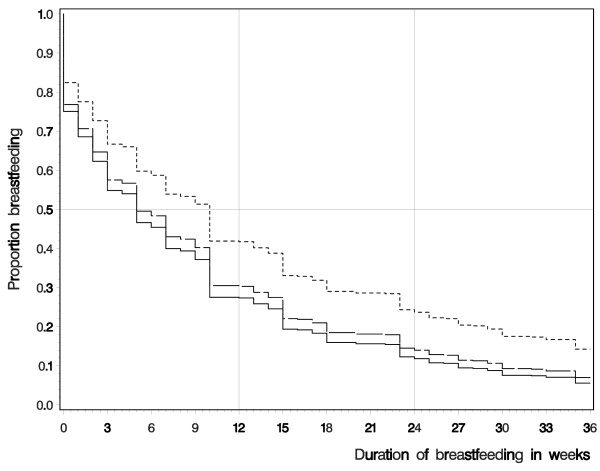
**Time windows of oral contraceptive (OC) use before conception:** ---------- No oral contraceptive use. ___ ___ ___  4-12 months prior conception. ___________  Less than 4 months prior conception.

Compared to those who did not use OC in the 12 months before pregnancy and controlling for confounders, mothers who used OC during the 3 months preceding conception had a shorter duration of breastfeeding (HR = 1.3; 95% CI: 1.03, 1.61; Table 3) with an adjusted median time of 5.47 weeks. Mothers who stopped OC use in the 4–12 months before conception (HR = 1.3, 95% CI: 1.02, 1.58) had an adjusted median duration of 6.13 weeks. Smoking during pregnancy was also related to shorter duration of breastfeeding (HR = 1.36; 95% CI: 1.13, 1.65); so was a lower education (HR = 1.4; 95% CI: 1.15, 1.71). Higher maternal education showed a statistically significant protective effect on breastfeeding duration (HR = 0.67; 95% CI: 0.53, 0.83). The proportion of women who continued breastfeeding shows that the difference among the three OC groups is established in the first few weeks and remains proportional thereafter (Figure 1).

**Table 3:** Relation between risk factors and breastfeeding duration (n = 563)

**Risk factors^#^**		**Hazard ratios (95% CI) (stopping breastfeeding)**	**p-value**
Oral contraceptives (months preceding conception)	None (reference)	1	
	0–3	1.29 (1.03, 1.61)	0.03
	4–12	1.27 (1.02, 1.58)	0.03
Mother age at pregnancy	20–24 years (reference)	1	
	< 20 years	1.11 (0.65, 1.91)	0.69
	25–29 years	0.93 (0.78, 1.11)	0.44
	≥ 30 years	4.11 (0.56, 29.80)	0.16
Maternal education	Some college (reference)	1	
	≤ High school	1.40 (1.15, 1.70)	< 0.01
	≥ College graduate	0.66 (0.53, 0.83)	< 0.01
Birth order	1	1.08 (0.90, 1.30)	0.38
	≥ 2 (reference)	1	
Smoke during pregnancy	No (reference)	1	
	Yes	1.36 (1.13, 1.65)	< 0.01
Age at menarche	≤ 11 years	1.00 (0.99, 1.00)	0.13
	12–15 years (reference)	1	
	≥ 16 years	1.00 (1.00, 1.01)	0.12
Planned pregnancy	No (reference)	1	
	Yes	1.06 (0.88, 1.29)	0.53

^#^Hazard ratios for specific risk factors were estimated controlling for all other independent variables presented in the table.

When stratifying for birth order (one, two and more), the HR for using OC during the 3 months preceding conception was stronger for the first pregnancy (HR = 1.38, 95% CI: 1.01, 1.87) than for pregnancies with a higher order (HR = 1.19, 95% CI: 0.84, 1.69; data not shown). Contrary to this finding, OC use in the 4–12 months before conception was no longer statistically significant (HR = 1.14, 95% CI: 0.86, 1.5) for the first pregnancy, but was important for later pregnancies (HR = 1.78, 95% CI: 1.23, 2.57). The association of maternal education and duration of breastfeeding did not change after stratification by birth order. Maternal smoking during pregnancy showed no statistically significant hazard ratio for breastfeeding after the first pregnancy (HR = 1.23; 95% CI: 0.89, 1.44), but an increased hazard ratio for smoking during pregnancy with higher parity (HR = 1.65, 95% CI: 1.22, 2.23). To stratify for planning this pregnancy, we grouped the variable into planning and no planning; the latter included undecided women and women with no intention to conceive. Interestingly, OC use showed no effect on duration of breastfeeding in women who did not plan their pregnancy or who were undecided. However, the associations between OC use and breastfeeding duration in women who planned their pregnancy did not differ from the findings in the total sample (Table 3). In other words, after stratifying for planning this pregnancy, the HR of stopping breastfeeding among those who used OC did not change.

When excluding non-breastfeeders from the analyses (excluding zero duration), only minor changes occurred in estimated hazard ratios (data not shown). The association between OC use during the 3 months preceding conception and breastfeeding duration became stronger (HR = 1.45, 95% CI: 1.27, 1.86), whereas the hazard ratio for OC use in the 4–12 months before conception did not substantially change (HR = 1.23 compared to HR = 1.27; Table 3).

## Discussion

Oral contraceptive use in the 12 months before pregnancy, smoking during pregnancy, and lower maternal education were related to a shorter duration of breastfeeding. To our knowledge no study has yet reported an association between use of OC before pregnancy and lower duration of breastfeeding.

One of the limitations of this study is the possibility of information bias as a result of recall bias, since the participants where asked about their reproductive history when the child was 7–9 years old. However, trained personnel conducted the interviews and helped to assess the most accurate time when the women started and stopped using OC. Nevertheless, some women might have forgotten the time when they started using OC and at what time they ceased. In addition, duration of breastfeeding may be subject to recall bias. However, studies have demonstrated that maternal recall of breastfeeding does not deteriorate substantially over time [22-25]. Nonetheless, there is no reason to assume that the recall of OC use varied in mothers with different durations of breastfeeding or vice versa. Hence, this non-differential misclassification is likely to underestimate the association between OC use and breastfeeding duration. Another limitation of this study is that we do not have data about some potential confounders such as type of OC, work status, Caesarean section, pacifier use, sore or bleeding nipple, and lack of partner support.

In this analysis, 575 of the 663 mothers participated in the reproductive interview and were included; 88 were not included, because of missing information or not being the biological mother. When comparing these two samples, there was no difference with regard to age and smoking during pregnancy (data not shown). However, participating women were more highly educated: 57% of the non-participants did not have high school education compared to only 35% of the participating women. Since the educational level was related to breastfeeding initiation, it is possible that the increased proportion of better educated women has augmented the proportion of breastfeeding women and the duration of breastfeeding. Such an educational difference is often seen in surveys [25]. However, it is unlikely that this selection affected the association between oral contraceptive use and breastfeeding duration, since non-OC use was more prevalent in women with college education (Table 1). There was also a significant difference in birth order distribution in the two samples: 54.4% of the participating women had one child whereas only 34.3% of the non-participating women had one child. Compared to women who did not use OC, the proportion of women who used oral contraceptives before their first pregnancy was higher (62.8% vs. 48.7%, Table 1). Hence it is possible that in our sample OC users were overrepresented due to more women with their first pregnancy. However, since birth order was not related to duration of breastfeeding (p = 0.36, Table 3), a potential over-representation of women who had one child should not have biased the association between OC use and breastfeeding duration. Also Shawky and Abalkhail reported that parity was not related to duration of breastfeeding [4].

The proportion of breastfeeding initiation identified in our study (81.4%) is comparable to findings by the German Health Interview and Examination Survey for Children and Adolescents. Across all age groups, 76.7% of German children were breastfed [26]. Based on another study, Bergmann and colleagues stated that 92% of their participants were ever breastfeed [27]. Kersting and Dulon conducted a cross sectional study to assess the breastfeeding promotion in hospitals in Germany [28]. They reported that 95% of the mothers start to breastfeed at birth. At the day of discharge, 86% were still breastfeeding. The proportion decreased to 56% at the age of 4 months. Our study shows a decrease of breastfeeding from 80% to 71% at 4 months.

Regarding education and smoking during pregnancy, our findings are in agreement with prior reports [3,8,26,29]. A German study reported that lack of breastfeeding was associated with lower maternal education [8]. In addition, the German Health Interview and Examination Survey showed that the proportion of ever-breastfed children was significantly lower in mothers from socially disadvantaged population groups [26]. Taylor and colleagues, using the 1995 National Survey of Family Growth in the United States, demonstrated that mothers who breastfed their children had more years of education than women who did not breastfeed [29]. This implies that education level may be a factor in breastfeeding initiation across Western cultures.

Only in women who planned their pregnancy (n = 421) the HRs for OC use in the 4–12 months before conception and OC use immediately before conception (0–3 months) were statistically significantly elevated. This finding suggests that the effect of OC use on the duration of breastfeeding is not explained by the status of pregnancy planning. On the contrary, the use of OC seems to be more frequent in women who planned their pregnancy. The reason may be that OC use provides a better control option. Hence, the stratification put forward the notion that the diminishing effect of OC use on duration of breastfeeding is not a shared intentional characteristic of a life style, but an unintentional effect.

Regarding smoking, in agreement with other studies we found that a lower proportion of mothers who had smoked during pregnancy initiated breastfeeding (Table 2) [3,8,26]. For a cohort of 1,098 Brazilian infants, Horta et al showed that maternal smoking reduced feeding duration [3]. A German study illustrated that breastfeeding duration of less than 4 months was associated with smoking during pregnancy [8]. In a sample of mothers from the United States, Vio et al reported that smoking during pregnancy reduces daily milk output by about 250–300 mL [30]. A potential explanation is an endocrine disrupting mechanism, namely that smoking increases dopamine secretion in the hypothalamus, which leads to a reduction in prolactin levels and thus reduced milk output [31].

A study from Saudi-Arabia showed that OC (type of OC not specified) use after delivery diminish the duration of breastfeeding [4]. Briend et al found that the use of a combined oral contraceptive (0.5 mg norgestrel and 0.05 ethinyl estradiol) significantly increased breastfeeding cessation [14]. Other studies stated that OC use shorten the duration of lactation and decrease human milk production [32,33]. Ingram and colleagues found a negative effect of higher estradiol levels at 4 weeks of lactation [34]. It has been suggested that estrogens block the action of prolactin on lactation [35,36]. In order to produce milk, prolactin is released. It then acts on human breast tissue to produce milk by binding to mammary epithelial cell receptors [37].

Our results add to the evidence that oral contraceptives may reduce breastfeeding duration. It is possible that oral contraceptive act via endocrine disruption. This proposition requires the initiation of long-term changes in sex steroid hormone levels by oral contraceptive use before pregnancy. In support of a long-term effect, Keski-Nisula et al showed that oral contraceptive use within one year before pregnancy increased estradiol (non-significant) and progesterone levels (statistically significant) during pregnancy [38]. Hence, it is biologically plausible that oral contraceptive use before pregnancy may affect hormone levels that then interfere with breastfeeding duration.

Our findings are in agreement with a prior report that endocrine disruption, for instance by DDE in maternal serum may be related to lower initiation rates and shorter duration of breastfeeding [10-12]. An alternate explanation is that both oral contraceptive use and a shorter duration of breastfeeding may represent lifestyle related conditions. For instance, women whose reproductive history is restricted by occupational or financial obligations may decide to plan their conception more carefully and may also reduce the duration of breastfeeding. The use of OC may give women more control over their lives and the risks of pregnancy and childbearing. Our results do not allow us to distinguish the effects of endocrine disruption or lifestyle choices in the association between oral contraceptive use before pregnancy and shorter duration of breastfeeding. However, to promote breastfeeding, there is a need to better understand which of the two mechanisms affect lactogenesis.

## Conclusion

These results suggest that in mothers who used oral contraceptives in the 12 months before conception, duration of breastfeeding was shorter compared to mothers who did not. Other risk factors for breastfeeding cessation were smoking during pregnancy and low level of maternal education. In the light of the established benefits of breastfeeding for mother-infant dyad, there is a need to better understand and prevent adverse effects of xenoestrogens and/or lifestyle choices on lactogenesis.

## Competing interests

The authors declare that they have no competing interests.

## Authors' contributions

NSR conducted data analysis and drafted the manuscript. WK was responsible for project design, data analysis and drafting of the manuscript.

## References

[B1] Victora CG, Smith PG, Vaughan JP, Nobre LC, Lombardi C, Teixeira AM, Fuchs SM, Moreira LB, Gigante LP, Barros FC (1987). Evidence for protection by breast-feeding against infant deaths from infectious diseases in Brazil. Lancet.

[B2] Grimes DA, Economy KE (1995). Primary prevention of gynecologic cancers. Am J Obstet Gynecol.

[B3] Horta BL, Victora CG, Menezes AM, Barros FC (1997). Environmental tobacco smoke and breastfeeding duration. Am J Epidemiol.

[B4] Shawky S, Abalkhail BA (2003). Maternal factors associated with the duration of breast feeding in Jeddah, Saudi Arabia. Paediatr Perinat Epidemiol.

[B5] Perez-Escamilla R, Maulen-Radovan I, Dewey KG (1996). The association between caesarean delivery and breast-feeding outcomes among Mexican women. Am J Public Health.

[B6] Coulibaly R, Seguin L, Zunzunegui MV, Gauvin L (2006). Links between maternal breast-feeding duration and Quebec infants' health: a population-based study. Are the effects different for poor children?. Matern Child Health J.

[B7] Wright CM, Parkinson K, Scott J (2006). Breast-feeding in a UK urban context: who breast-feeds, for how long and does it matter?. Public Health Nutr.

[B8] Kohlhuber M, Rebhan B, Schwegler U, Koletzko B, Fromme H (2008). Breastfeeding rates and duration in Germany: a Bavarian cohort study. Br J Nutr.

[B9] Hawkins SS, Griffiths LJ, Dezateux C, Law C (2007). Maternal employment and breast-feeding initiation: findings from the Millennium Cohort Study. Paediatr Perinat Epidemiol.

[B10] Rogan WJ, Gladen BC, McKinney JD, Carreras N, Hardy P, Thullen J, Tingelstad J, Tully M (1987). Polychlorinated biphenyls (PCBs) and dichlorodiphenyl dichloroethene (DDE) in human milk: effects on growth, morbidity, and duration of lactation. Am J Public Health.

[B11] Gladen BC, Rogan WJ (1995). DDE and shortened duration of lactation in a northern Mexican town. Am J Public Health.

[B12] Karmaus W, Davis S, Fussman C, Brooks K (2005). Maternal concentration of dichlorodiphenyl dichloroethylene (DDE) and initiation and duration of breast feeding. Paediatr Perinat Epidemiol.

[B13] US Environmental Protection Agency (1998). Endocrine Disruptor Screening and Testing Advisory Committee (EDSTAC) Final Report Washington DC.

[B14] Briend A, Fauveau V, Chakraborty J (1991). Contraceptive use and breast-feeding duration in rural Bangladesh. Eur J Clin Nutr.

[B15] Osius N, Karmaus W, Kruse H, Witten J (1999). Exposure to polychlorinated biphenyls and levels of thyroid hormones in children. Environ Health Perspect.

[B16] Karmaus W, Brooks KR, Nebe T, Witten J, Obi-Osius N, Kruse H (2005). Immune function biomarkers in children exposed to lead and organochlorine compounds: a cross-sectional study. Environ Health.

[B17] Karmaus W, DeKoning EP, Kruse H, Witten J, Osius N (2001). Early childhood determinants of organochlorine concentrations in school-aged children. Pediatr Res.

[B18] Karmaus W, Kuehr J, Kruse H (2001). Infections and atopic disorders in childhood and organochlorine exposure. Arch Environ Health.

[B19] Karmaus W, Davis S, Chen Q, Kuehr J, Kruse H (2003). Atopic manifestations, breast-feeding protection and the adverse effect of DDE. Paediatr Perinat Epidemiol.

[B20] Karmaus W, Juul S (1999). Infertility and subfecundity in population-based samples from Denmark, Germany, Italy, Poland and Spain. Eur J of Public Health.

[B21] SAS-Institute (2006). SAS/STAT Software, v.9.1.

[B22] Tomeo CA, Rich-Edwards JW, Michels KB, Berkey CS, Hunter DJ, Frazier AL, Willett WC, Buka SL (1999). Reproducibility and validity of maternal recall of pregnancy-related events. Epidemiology.

[B23] Gillespie B, d'Arcy H, Schwartz K, Bobo JK, Foxman B (2006). Recall of age of weaning and other breastfeeding variables. Int Breastfeed J.

[B24] Li R, Scanlon KS, Serdula MK (2005). The validity and reliability of maternal recall of breastfeeding practice. Nutr Rev.

[B25] Tolonen H, Helakorpi S, Talala K, Helasoja V, Martelin T, Prattala R (2006). 25-year trends and socio-demographic differences in response rates: Finnish adult health behaviour survey. Eur J Epidemiol.

[B26] Lange C, Schenk L, Bergmann R (2007). [Distribution, duration and temporal trend of breastfeeding in Germany. Results of the German Health Interview and Examination Survey for Children and Adolescents (KiGGS)]. Bundesgesundheitsblatt Gesundheitsforschung Gesundheitsschutz.

[B27] Bergmann RL, Diepgen TL, Kuss O, Bergmann KE, Kujat J, Dudenhausen JW, Wahn U (2002). Breastfeeding duration is a risk factor for atopic eczema. Clin Exp Allergy.

[B28] Kersting M, Dulon M (2002). Assessment of breast-feeding promotion in hospitals and follow-up survey of mother-infant pairs in Germany: the SuSe Study. Public Health Nutr.

[B29] Taylor JS, Risica PM, Cabral HJ (2003). Why primiparous mothers do not breastfeed in the United States: a national survey. Acta Paediatr.

[B30] Vio F, Salazar G, Infante C (1991). Smoking during pregnancy and lactation and its effects on breast-milk volume. Am J Clin Nutr.

[B31] Jansson A, Andersson K, Bjelke B, Eneroth P, Fuxe K (1992). Effects of a postnatal exposure to cigarette smoke on hypothalamic catecholamine nerve terminal systems and on neuroendocrine function in the postnatal and adult male rat. Evidence for long-term modulation of anterior pituitary function. Acta Physiol Scand.

[B32] Kora SJ (1969). Effect of oral contraceptives on lactation. Fertil Steril.

[B33] Nilsson S, Mellbin T, Hofvander Y, Sundelin C, Valentin J, Nygren KG (1986). Long-term follow-up of children breast-fed by mothers using oral contraceptives. Contraception.

[B34] Ingram JC, Woolridge MW, Greenwood RJ, McGrath L (1999). Maternal predictors of early breast milk output. Acta Paediatr.

[B35] Truitt ST, Fraser AB, Grimes DA, Gallo MF, Schulz KF (2003). Hormonal contraception during lactation. systematic review of randomized controlled trials. Contraception.

[B36] Nedkova V, Tanchev S (1995). [Serum levels of prolactin, progesterone and estradiol in nursing mothers]. Akush Ginekol (Sofiia).

[B37] Lawrence RA, Lawrence RM (2005). Breastfeeding, A Guide for the Medical Profession.

[B38] Keski-Nisula L, Pekkanen J, Xu B, Putus T, Koskela P (2006). Does the pill make a difference? Previous maternal use of contraceptive pills and allergic diseases among offspring. Allergy.

